# MiR-320a induces diabetic nephropathy via inhibiting MafB

**DOI:** 10.18632/aging.101962

**Published:** 2019-05-17

**Authors:** Mengying He, Jin Wang, Zhongwei Yin, Yanru Zhao, Huiying Hou, Jiahui Fan, Huaping Li, Zheng Wen, Jiarong Tang, Yan Wang, Dao Wen Wang, Chen Chen

**Affiliations:** 1 Division of Cardiology and Hubei Key Laboratory of Genetics and Molecular Mechanisms of Cardiological Disorders, Tongji Hospital, Tongji Medical College, Huazhong University of Science and Technology, Wuhan 430030, China

**Keywords:** miRNA, podocyte, diabetic nephropathy

## Abstract

Multiple studies indicate that microRNAs (miRNAs) are involved in diabetes. However, the roles of miRNA in the target organ damages in diabetes remain unclear. This study investigated the functions of miR-320a in diabetic nephropathy (DN). In this study, db/db mice were used to observe the changes in podocytes and their function in vivo, as well as in cultured mouse podocyte cells (MPC5) exposed to high glucose in vitro. To further explore the role of miR-320a in DN, recombinant adeno-associated viral particle was administered intravenously to manipulate the expression of miR-320a in db/db mice. Overexpression of miR-320a markedly promoted podocyte loss and dysfunction in DN, including mesangial expansion and increased levels of proteinuria, serum creatinine and urea nitrogen. Furthermore, MafB was identified as a direct target of miR-320a through AGO2 co-immunoprecipitation, luciferase reporter assay, and Western blotting. Moreover, re-expression of MafB rescued miR-320a-induced podocyte loss and dysfunction by upregulating the expressions of Nephrin and glutathione peroxidase 3 (Gpx3). Our data indicated that miR-320a aggravated renal disfunction in DN by targeting MafB and downregulating Nephrin and Gpx3 in podocytes, which suggested that miR-320a could be a potential therapeutic target of diabetic nephropathy.

## INTRODUCTION

Diabetes is one of the most common chronic diseases worldwide [1]. Recently, more and more attentions focused on diabetes induced severe complications such as neuropathy, retinopathy, nephropathy and cardiovascular diseases. Two of ten patients with diabetes, either type 1 or type 2, will develop diabetic nephropathy (DN) after 10 to 20 years, which makes diabetes the main cause of end stage renal diseases in western societies [2]. While 10 to 40 percent of patients with type 2 diabetes finally develop DN in urban China [3, 4]. It seems that all types of the kidney cells, including glomerular epithelial (podocyte), endothelial (GECs) and mesangial cells, tubular epithelia, vascular endothelia and interstitial fibroblasts are sensitivity to hyperglycemia in varying degrees. The dysfunctions of the glomerular filtration barrier, which comprises GECs separated from podocytes by the glomerular basement membrane (GBM) may lead to albuminuria [5], an increase of urinary protein, which not only is an early sign of diabetic nephropathy, but also can predict the progression of renal damage [6].

Mature podocytes are terminally differentiated epithelial cells that cover the outer side of the GBM, consisting of a large cell body, major processes and foot processes [7, 8]. Differently from cell bodies and major processes floating freely in Bowman’s space, foot processes are situated on the GBM, which ultimately form a unique interdigitating pattern with neighboring cells [8, 9]. Adjacent FPs are connected by the glomerular slit diaphragm which is regarded as the main size selective filter barrier and composed of proteins including Nephrin, podocin, P-cadherin, CD2AP, etc [8]. Podocytes are key components of the selective permeability barrier of the GBM, and diabetes induced apoptosis of podocytes contributed to the dysfunctions of the glomerular filtration barrier, which may lead to albuminuria [10]. Meanwhile, podocyte deletion can arise in the early stage of DN and predict the clinical progression [11]. Recently, it was found that enhanced oxidative stress may lead to podocyte loss [12]. However, the underlying specific mechanisms were still unrevealed.

MafB is a member of the large Maf family, which contains a basic leucine zipper that mediates dimer formation and target DNA binding to the Maf recognition element (MARE) [13]. Previous researches have showed that MafB played a vital role in podocyte differentiation and its foot process formation [14]. MafB-deficient mice would die during the perinatal period [15]. Mutations of MafB impaired development and maintenance of podocytes [16], which resulted in focal segmental glomerulosclerosis (FSGS) [16] and carpotarsal osteolysis (MCTO) [17]. FSGS is a leading cause of end-stage renal diseases in children and adults [16], while MCTO is a rare skeletal dysplasia frequently associated with progressive renal failure [17]. MafB gene has been identified in the vicinity of the susceptible locus for albuminuria by linkage analysis in diabetic KKT/a mice, and its expression were decreased significantly in the diabetic kidneys [18]. A recent study showed that overexpression of MafB in podocytes prevented the development of diabetic nephropathy [19].

MicroRNAs (miRNAs) are short (usually about 22 nucleotides) noncoding RNAs, which regulate gene expression by inducing degradation or translational repression of target mRNA in animals and plants [20, 21]. MiRNAs generally bind to complementary sites within the 3′ UTRs of their target mRNAs incompletely [22, 23]. Recent studies have demonstrated that miRNAs not only played important roles in various biological processes, such as development and differentiation, but also acted as biomarkers in multiple human diseases [24, 25]. Among them, increased miR-320a was found in the plasma of patients with diabetes or diabetic animal models by investigating the miRNAs profiles in diabetes [26–28]. Most importantly, a cohort study revealed that the increased circulating level of miR-320a was a consequence of diabetic kidney dysfunction, and would be restored to normal level after simultaneous pancreas-kidney transplantation [26, 27]. Moreover, miR-320a promoted insulin resistance in high glucose treated adipocytes [29], and impaired myocardial microvascular angiogenesis in type 2 diabetic Goto-Kakizaki rats [28]. Our previous study also showed that the levels of circulating miR-320a was elevated in patients with coronary artery disease (CAD) and high-risk individuals of CAD, including persons with diabetes [30]. Meanwhile, we discovered that miR-320a contributed to metabolism disorder associated injury. However, whether miR-320a participates in diabetic kidney dysfunction is still unknown. Thus, we investigated the role of miR-320a in DN and the underlying mechanisms in the current study.

## RESULTS

### MiR-320a level was increased in the kidney of diabetic mice

**Figure 1:**
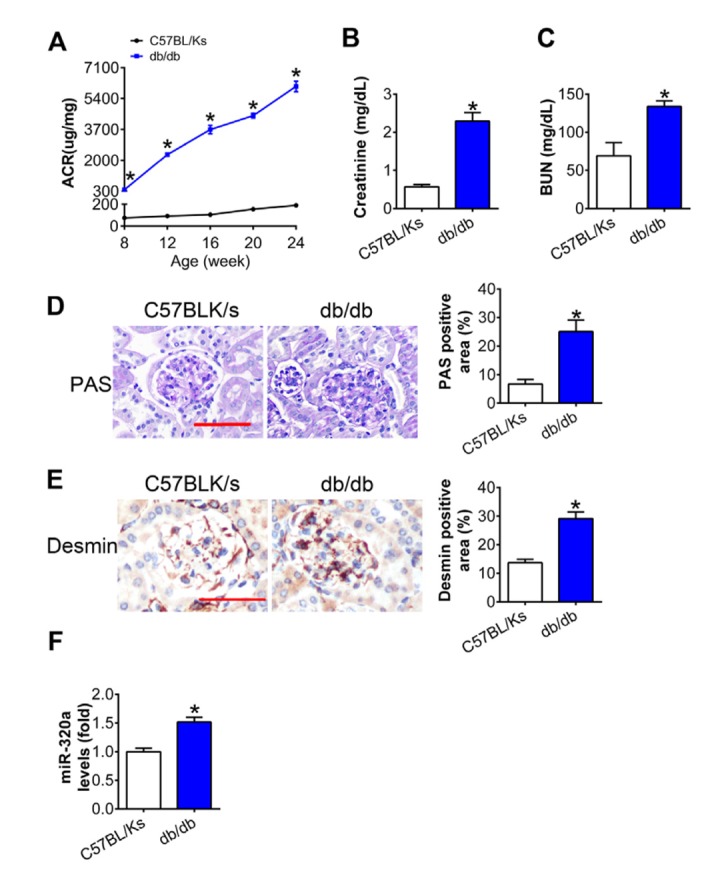
**MiR-320a was increased in the kidney of diabetic mice.** (**A**) Urinary ACR was determined every four weeks since the age of 8 weeks. (**B**) Serum creatinine and (**C**) BUN were detected at the age of 24 weeks. (**D**) Representative images of PAS staining of kidneys from C57BL/Ks and db/db mice. Scale bar, 50 μm. (**E**) Representative images of immunohistochemical staining of Desmin. Scale bar, 50 μm. (**F**) Relative miR-320a expression in renal cortex measured by real-time PCR. Data are expressed as mean ± SEM, n=8, *P<0.05 versus C57BL/Ks.

As shown in Figure 1A, albumin-to-creatinine ratio (ACR) was gradually elevated with age in db/db mice compared with C57BL/Ks mice, which indicated diabetic kidney dysfunction. Further, the levels of serum creatinine (CR) and blood urea nitrogen (BUN) in db/db mice were remarkedly increased compared with C57BL/Ks mice at the age of 24 weeks (Figure 1B and 1C). Moreover, periodic acid–Schiff (PAS) staining revealed mesangial expansion in the kidney of db/db mice (Figure 1D). Meanwhile, immunostaining of Desmin, the podocyte injury marker, indicated podocyte injury in diabetic glomeruli (Figure 1E). By using quantitative RT-PCR assays, increased miR-320a was found in the renal cortex of db/db mice compared to C57BL/Ks mice (Figure 1F). These results suggested that db/db mice developed DN, and miR-320a might participate in the pathological process of DN.

### Overexpression of miR-320a aggravated renal dysfunction in db/db mice

**Figure 2:**
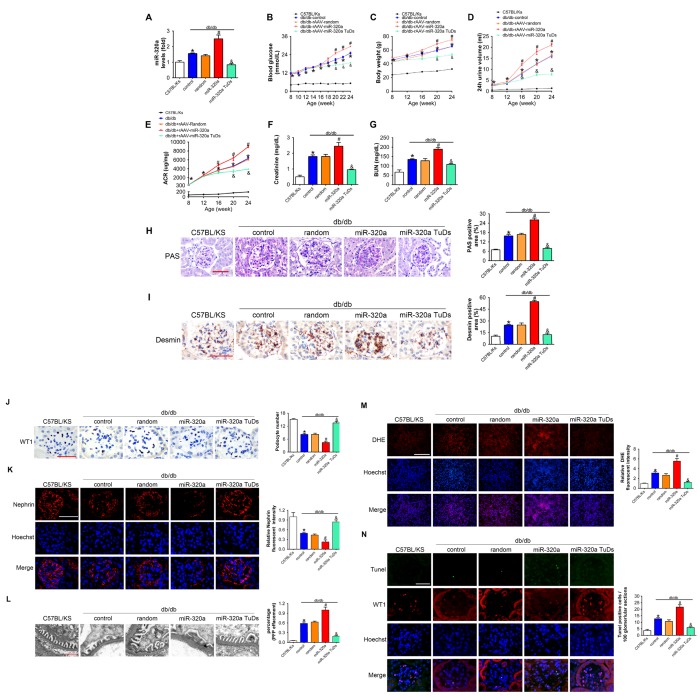
**Overexpression of miR-320a aggravated renal dysfunction in db/db mice.** (**A**) Relative miR-320a expression in renal cortex measured by real-time PCR. (**B**) Blood glucose was detected every 2 weeks. (**C**) Body weight, (**D**) 24h urine volume and (**E**) urinary ACR was determined every four weeks since the age of 8 weeks. (**F**) Serum creatinine and (**G**) BUN were detected at the age of 24 weeks. (**H**) Representative images of PAS staining of kidneys from C57BL/Ks and differently treated db/db mice. Scale bar, 50 μm. (**I**) Representative images of immunohistochemical staining of Desmin. Scale bar, 50 μm. (**J**) Typical images of WT1-stained glomeruli and average number of WT1-stained nuclei calculated per glomerular sections. Scale bar, 50 μm. (**K**) Representative images of immunofluorescence staining for Nephrin. Scale bar, 50 μm. (**L**) Representative electron microscopic image of the glomeruli staining from kidney sections. Scale bar, 1 μm. (**M**) Representative images of ROS detected by DHE probe in frozen kidney sections. Scale bar, 200 μm. (**N**) Typical images of apoptotic glomerular cells in diabetic glomeruli. Green, TUNEL; Red, WT1; Blue, Hoechst. Scale bar, 50 μm. Data are expressed as mean ± SEM, n=8, *P<0.05 versus C57BL/Ks, #P<0.05 versus db/db control, &P<0.05 versus db/db control.

In order to explore the effects of miR-320a in DN, recombinant adeno-associated viral (rAAV) system was used to manipulate the expression levels of mature miR-320a in mice. We found that almost all karyotes in glomeruli and the majority of tubular epithelial cells were efficiently transfected, while the fluorescence intensity of the GFP staining in the pancreas was limited (Supplementary Figure 1A and 1B). After 4 months, treated db/db mice were sacrificed and it was found that rAAV-miR-320a treatment elevated the level of miR-320a, while rAAV-miR-320a TuDs reduced the expression of miR-320a in the renal cortex of db/db mice (Figure 2A). During the observation period, the level of blood glucose and body weight were elevated in db/db mice compared with control mice. Among them, db/db mice treated with rAAV-miR-320a had increased blood glucose and weight gain than the control db/db mice, while db/db mice with rAAV-miR-320a TuDs exhibited opposite effects (Figure 2B and 2C). Of note, miR-320a overexpression exacerbated the diabetes-induced renal dysfunction as evidenced by increased 24h urine volume, ACR, serum CR and BUN (Figure 2D–2G). On the contrary, knockdown of miR-320a by rAAV-miR-320a TuDs attenuated the renal dysfunction in db/db mice (Figure 2D–2G). Furthermore, overexpression of miR-320a increased diabetes-induced mesangial expansion and podocyte injury in diabetic glomeruli compared with control db/db mice, while knockdown of miR-320a alleviated these injuries (Figure 2H and 2I). These data indicated that miR-320a damaged the integrity of GBM in db/db mice.

As proteinuria is often related to podocyte loss or dysfunction [11], we then measured the morphology and function of podocyte. We found that podocyte architectural integrity was disrupted in diabetic glomeruli as evidenced by the loss of WT1-positive podocytes, decreased Nephrin expression and effaced podocyte foot processes under electron microscope (Figure 2L–2L). Whereas these changes were mitigated by rAAV-miR-320a TuDs (Figure 2J–2L). Podocytes are susceptible to the damage of oxidative stress [6, 12], and podocyte loss is largely attributed to apoptosis in kidney diseases [12, 31]. Therefore, oxidative stress in kidney was evaluated using Dihydroethidium (DHE) probe. It was found that overexpression of miR-320a increased ROS in kidney of db/db mice, while knockdown of miR-320a reversed diabetes-induced ROS activation (Figure 2M). Further, the kidney sections were subjected to TUNEL staining, and the results showed that rare apoptotic glomerular cells were observed in kidneys of C57BL/Ks mice (Figure 2N). However, the number of WT1 and TUNEL-double positive glomerular cells, which indicated apoptotic podocytes, significantly increased in kidney of db/db mice (Figure 2N). Moreover, miR-320a overexpression promoted apoptosis of podocytes in diabetic kidney, while knockdown of miR-320a alleviated podocyte apoptosis (Figure 2N). Additionally, we found no significant difference among normal C57BL/Ks mice with different treatments (Supplementary Figure 2A–2G), which indicated that miR-320a did not damage kidney function and podocytes under normoglycemic condition.

### Overexpression of miR-320a enhanced hyperglycemia induced podocytes injury in vitro

**Figure 3:**
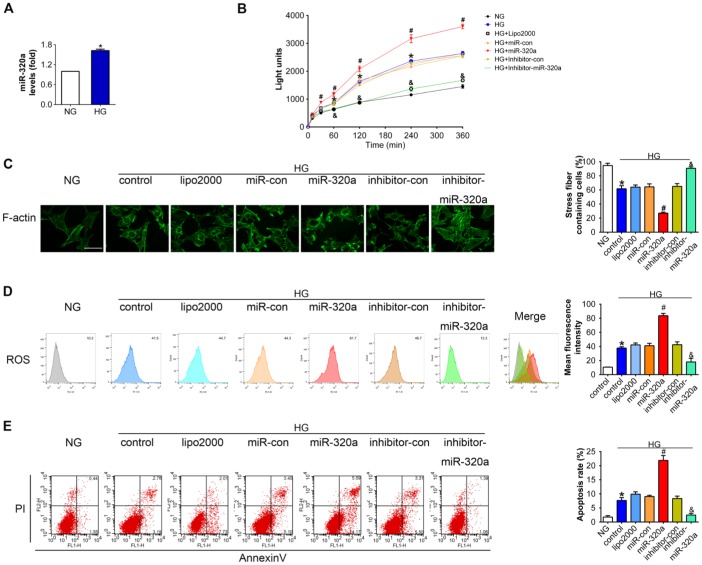
**Overexpression of miR-320a enhanced hyperglycemia induced podocytes injury in vitro.** (**A**) Relative miR-320a expression in cultured podocyte cells exposed to normal glucose (NG, 5 mM) and high glucose (HG, 30 mM). (**B**) In vitro permeability of FITC-labeled BSA through podocyte monolayers. (**C**) Representative photomicrographs of immunofluorescence labeling with F-actin in cultured podocyte cells. Effects of miR-320a mimics on apoptosis (**D**) and ROS (**E**) were determined by Annexin V/PI flow cytometric analysis and DHE in cultured podocyte cells. Data are representative of three experiments. Data are expressed as mean ± SEM, n=3, *P<0.05 versus NG, #P<0.05 versus HG + miR-con, &P<0.05 versus HG + inhibitor-con.

To further investigate the effects of miR-320a in podocytes, in vitro studies were performed using miR-320a mimics/inhibitor transfection in cultured murine podocytes. Podocytes were cultured in medium with high glucose (HG, 30 mM) for 48 h to simulate a cell model of hyperglycemia. We found that the level of miR-320a was increased in HG-treated podocytes compared with normal glucose (NG) (Figure 3A). Meanwhile, overexpression of miR-320a caused an increase in albumin permeability in cultured podocytes with HG treatment, suggesting worse podocyte architectural integrity (Figure 3B). Moreover, overexpression of miR-320a resulted in enhanced reorganization of actin cytoskeleton in podocytes, compared with control group (Figure 3C). Besides, significant increase in ROS and apoptosis were observed in HG treated podocytes by miR-320a mimics transfection, while miR-320a inhibitor alleviated these effects of HG (Figure 3D and 3E).

### MafB is a target of miR-320a

**Figure 4:**
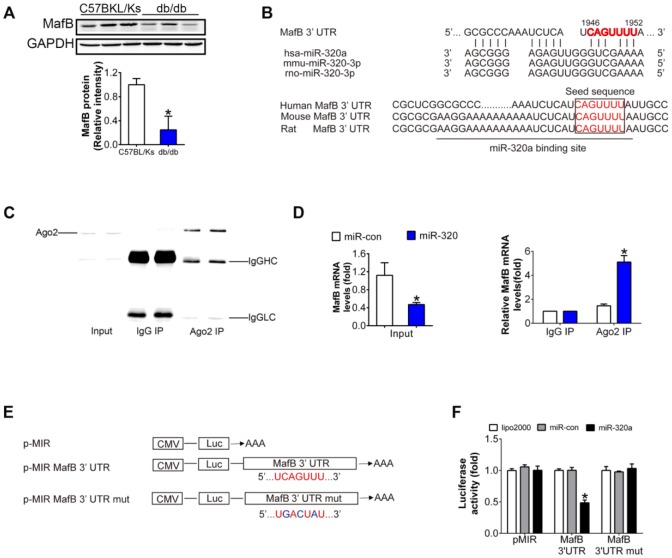
**MafB is a target of miR-320a.** (**A**) MafB protein levels detected by western blot in C57BLKS and db/db mice. (**B**) miR-320a and the 3’-UTR of MafB among three species. (**C**) Ago2 protein levels in co-immunoprecipitated products detected by Western blot. IgGHC, IgG heavy chain; IgGLC, IgG light chain. (**D**) Relative expression of MafB in the whole RNA (left) and RNA of the nonspecific IgG or anti-Ago2 co-IP (right) from the HG-treated podocyte cell lysates. #P<0.05 versus miR-con + input, *P<0.05 versus miR-con + IgG IP. (**E**) Schematic diagram of the luciferase reporter plasmids of pMIR-MafB 3’-UTR and pMIR-MafB 3’-UTR mut, and the potential target site of miR-320a on the 3’-UTR of MafB. (**F**) Regulation of miR-320a on 3’-UTR of MafB in HEK293 cells by luciferase reporter assay. *P<0.05 versus MafB 3’-UTR + miR-con.

A series of genes were down-regulated in glomeruli of diabetic mice according to the microarray data deposited in Gene Expression Omnibus (accession number GSE20844) (Supplementary Table 1). Among them, 3 were predicted targets of miR-320a by minimum free energy (MFE) (≤ -25 kcal/mol) calculation and conversation among species, while only MafB was obviously downregulated in diabetic kidney (Figure 4A and 4B, Supplementary Figure 3). MafB played an important role in renal diseases as reported previously [14]. Multiple sequence alignment of hsa-miR-320a indicated a highly conserved binding site of miR-320a within the 3′ UTR of MafB gene among different species (Figure 4B). To validate whether MafB was a functional target of miR-320a in DN, we immunoprecipitated Argonaute 2 (Ago2), an important element of RNA-induced silencing complex (RISC), from HG-treated podocyte cell lysates. The results showed that Ago2 was specifically isolated with the anti-Ago2 antibody not with nonspecific IgG (Figure 4C). Meanwhile, Ago2 showed increased association with the MafB mRNA after miR-320a mimics transfection (Figure 4D). Moreover, luciferase reporter assays were performed to identify the specific binding site of miR-320a in MafB 3′ UTR (Figure 4E). When co-transfected with miR-320a mimics, the relative luciferase activity of MafB 3′ UTR reporter was obviously suppressed compared with the transfection of miR-con as well as with empty vector or the mutant reporter. (Figure 4F).

### Overexpression of miR-320a down-regulated MafB in vitro and in vivo

**Figure 5:**
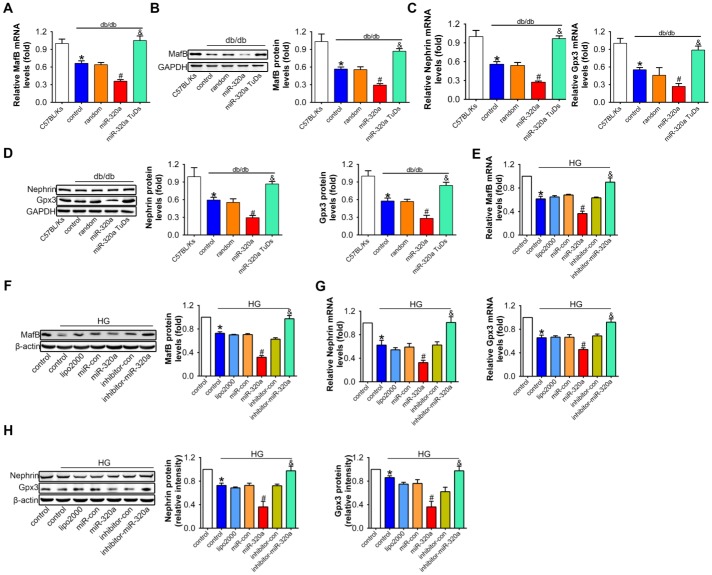
**Overexpression of miR-320a down-regulated MafB in vitro and in vivo.** Relative MafB (**A**) mRNA and (**B**) protein levels in differently treated db/db mice. Relative Nephrin and Gpx3 (**C**) mRNA and (**D**) protein expression in differently treated db/db mice. Data are expressed as mean ± SEM, n=8, *P<0.05 versus C57BL/Ks, #P<0.05 versus db/db control, &P<0.05 versus db/db control. Relative MafB (**E**) mRNA and (**F**) protein levels in cultured podocyte cells. Relative Nephrin and Gpx3 expression measured by (**G**) real-time PCR and detected by (**H**) western blot. Data are expressed as mean ± SEM, n=3, *P<0.05 versus NG, #P<0.05 versus HG + miR-con, &P<0.05 versus HG + inhibitor-con.

The analysis of real-time PCR and Western blots showed that comparing with control db/db mice, mRNA and protein levels of MafB were both further reduced in rAAV-miR-320a treated db/db mice, while rAAV-miR-320a TuDs treatment showed opposite effects (Figure 5A and 5B).

To investigate whether the effects of miR-320a on MafB expression was related with podocyte loss in glomeruli, we additionally performed a two-week-study in normal C57BL/Ks mice using the rAAV system. Results showed that the value of blood glucose, body weight, urinary ACR, serum CR and BUN did not change among different groups (Supplementary Figure 4A–4F). Meanwhile, rAAV-miR-320a increased the level of miR-320a but slightly down-regulated MafB in the kidney (Supplementary Figure 4G–4I). More important, the unchanged WT1 staining among normal C57BL/Ks mice with different treatments indicated that the number of podocytes in the glomeruli was not affected by miR-320a (Supplementary Figure 4J). Together, these data indicated that miR-320a directly inhibited the expression of MafB.

To further explore the role of miR-320a/MafB signal in podocytes injury, the downstream signals genes of MafB were detected, for example, Nephrin and Gpx3, which were produced by podocytes in the glomeruli [32, 33]. Our results showed that mRNA and protein levels of Nephrin and Gpx3 both decreased in miR-320a overexpressed db/db mice, compared with control db/db mice (Figure 5C and 5D).

Consistently, in cultured podocytes, miR-320a mimic transfection significantly reduced MafB level, and miR-320a inhibitor increased MafB level (Figure 5E and 5F). Moreover, the mRNA and protein expression of Nehrin and Gpx3 were further decreased in HG-treated compared with the transfection of miRNA control (Figure 5G and 5H).

### MafB restoration attenuated miR-320a induced kidney injury in diabetes

**Figure 6:**
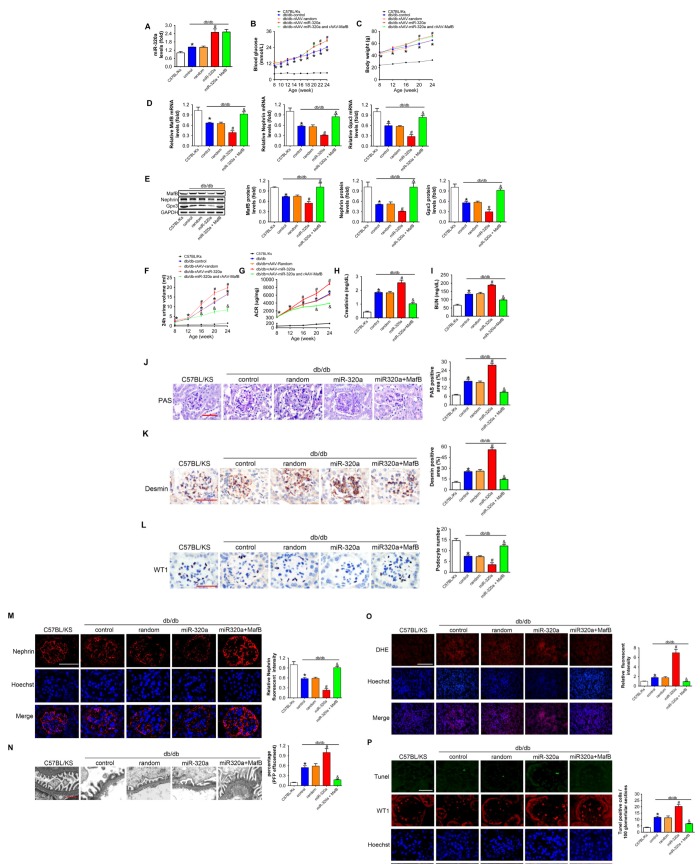
**MafB restoration attenuated miR-320a induced kidney injury in diabetes.** (**A**) Relative miR-320a expression in renal cortex measured by real-time PCR. (**B**) Blood glucose was detected every 2 weeks. (**C**) Body weight was measured once per month. Relative mRNA (**D**) and protein (**E**) levels of Mafb, Nephrin and Gpx3. (**F**) 24h urine volume and (**G**) urinary ACR was determined every four weeks since the age of 8 weeks. (**H**) Serum creatinine and (**I**) BUN were detected at the age of 24 weeks. (**J**) Representative images of PAS staining of kidneys from C57BL/Ks and differently treated db/db mice. Scale bar, 50 μm. (**K**) Representative images of immunohistochemical staining of Desmin. Scale bar, 50 μm. (**L**) Typical images of WT1-stained glomeruli and average number of WT1-stained nuclei calculated per glomerular sections. Scale bar, 50 μm. (**M**) Representative images of immunofluorescence staining for Nephrin. Scale bar, 50 μm. (**N**) Representative electron microscopic image of the glomeruli staining from kidney sections. Scale bar, 1 μm. (**O**) Representative images of ROS detected by DHE probe in frozen kidney sections. Scale bar, 200 μm. (**P**) Typical images of apoptotic glomerular cells in diabetic glomeruli. Green, TUNEL; Red, WT1; Blue, Hoechst. Scale bar, 50 μm. Data are expressed as mean ± SEM, n=8, *P<0.05 versus C57BL/Ks, #P<0.05 versus db/db control, &P<0.05 versus db/db control.

To verify the role of miR-320a/MafB signal in DN, we re-expressed MafB in rAAV-miR-320a-treated db/db mice using rAAV system. The results showed that rAAV-miR-320a significantly increased the level of miR-320a in the kidney of db/db mice (Figure 6A). Moreover, mice treated with rAAV-miR-320a exhibited increased blood glucose and weight gain than the control group, while rAAV-MafB showed no effects in db/db mice (Figure 6B and 6C). Verified by real-time PCR and Western blots, rAAV-MafB restored MafB expression in rAAV-miR-320a-treated db/db mice (Figure 6D and 6E). Consistently, restoration of MafB increased the levels of Nephrin and Gpx3 (Figure 6D and 6E), and reversed the miR-320a induced injury in diabetic kidney, as determined by 24h urine volume, urinary ACR, serum CR, BUN, glomerular PAS staining and immunostaining of Desmin (Figure 6F–6K). Meanwhile, rAAV-MafB mitigated the loss of WT1-positive podocytes, decreased Nephrin and effaced podocyte foot processes in miR-320a treated db/db mice (Figure 6L–6N). In addition, miR-320a increased the ROS level and apoptosis of podocytes, while restored MafB attenuated these effects (Figure 6O and 6P).

## DISCUSSION

In the present study, we showed that miR-320a aggravated kidney dysfunction in DN by suppressing MafB in podocytes. MiR-320a induced podocyte injury, which in turn led to the destraction of podocyte architectural integrity and mesangial expansion, and finally aggravated kidney dysfunction in diabetes.

With deficiency of leptin receptor expression, db/db mice from C57BL/KsJ background exhibit progressive obese, hyperglycemia and hyperinsulinemia from a very young age, which make them an eligible obese and type 2 diabetic mouse model [34]. Because of consistent and seriously elevated albuminuria and mesangial expansion in kidney, db/db mice most closely simulate the natural progression of human DN, comparing with various diabetic mouse models [35]. Therefore, in the current study, db/db mice were used as animal model of DN, which manifested progressive proteinuria, poor renal function, and apparent mesangial expansion.

Epidemiologic studies revealed that the increase of miR-320a in patients or animal models with diabetes was correlated with poor progression [26–28]. It was found that miR-320a promoted insulin resistance in high glucose treated adipocytes [29], and impaired myocardial microvascular angiogenesis of type 2 diabetic Goto-Kakizaki rats [28]. Our previous studies also showed that the levels of circulating miR-320a was elevated in patients with coronary artery disease (CAD) and high-risk individuals of CAD, including persons with diabetes [30]. All these suggested that miR-320a may participate in the multiple end organ damages of diabetes, but the biologic role of the miR-320a in DN remained unclear. In the current study, expression of miR-320a increased in both HG-treated podocyte cells and kidney of db/db mice. Moreover, overexpression of miR-320a in db/db mice presented more aggressive albuminuria, worse renal dysfunction and mesangial expansion than those untreated control db/db mice, while these changes were alleviated by rAVV-miR-320a TuDs.

To verify whether the resulting dysfunctions are also detectable in the normoglycemic mice, we performed the same experiments in normal C57BL/Ks mice. We found that overexpression of miR-320a did not cause damage to podocytes under normal circumstances, indicating that miR-320a-related podocyte injury and kidney dysfunction were specific to the diabetic animals.

Previously, VEGFA was described as a target gene down-regulated by miR-320a in the heart [36]. It is well known that VEGFA is highly expressed in podocytes from the glomeruli [37] and provides essential signals to maintain the function of glomerular endothelial cells including survival, regeneration [38], fenestrations [39] and ultrafiltration coefficient [40, 41]. It has been reported that blockade of VEGFA in some experimental animal models reduced vascular permeability [42, 43], including proteinuria in DN [44, 45]. Moreover clinical inhibition of VEGFA with anti-VEGFA antibodies [46] could cause proteinuria and hypertension in human [47], confirming that certain amount of VEGFA is necessary for endothelial maintenance. We have detected the level of VEGFA in the kidney of db/db mice with different treatments via western blot. The results showed that VEGFA was downregulated in db/db control mice compared with C57BL/Ks mice, while rAAV-miR-320a didn’t enhance the decrease of VEGFA in db/db mice (Supplementary Figure 5). It is also well known that one certain miRNA may target different molecular under various conditions, while one molecular maybe targeted by different miRNAs under various conditions. For example, miR-21 has been reported to aggravate glomerulosclerosis in diabetic nephropathy by inhibiting matrix metalloproteinases (MMP)-9 [48]. While both miR-21 [49] and MMP-9 [50] were increased in the heart, which promoted cardiac dysfunction and fibroblast in cardiac hypotrophy mouse model, suggesting that MMP-9 was not regulated by miR-21 in this model. Therefore, VEGFA may not account for the miR-320a related-glomerular dysfunction in the current study.

The adeno-associated virus (AAV) appears to be the most advantageous for its efficient transduction, long-term gene expression, low immunogenicity and lack of apparent cytotoxicity in tissues [51, 52]. Moreover, rAAV9 is reported as an efficacious serotype for kidney gene transfer [53]. In our study, rAVV9 was applied intravenously to manipulate miR-320a signaling expression in renal microenvironments. Interestingly, the blood glucose and weight gain were increased in db/db mice treated with rAAV-miR-320a comparing to control db/db mice, while restoration of MafB alleviated miR-320a induced podocyte injury and renal dysfunction without affecting blood glucose and body weight. In addition, it has been reported that miR-320a was presented in exosome [54]. As we also detected an increase of circulating miR-320a in db/db mice (Supplementary Figure 6), it was possible that circulating miR-320a may exert distant effects. In the current study, we mainly focused on the local effects of miR-320a in the kidney. We will explore the distant effects of miR-320a using co-culture in vitro and transplantation in vivo in the future.

Loss of podocytes contributes to the progression of diabetic nephropathy [55]. It was reported that podocytes were susceptible to oxidative stress, which may lead to podocyte deletion, in part, by apoptosis. On the other hand, oxidative stress was identified as a key initiator of the pathogenesis of DN [56, 57]. In addition, antioxidant showed potential beneficial effects in experimental diabetic nephropathy [57–59]. Our data showed that overexpression of miR-320a promoted the diabetes induced ROS production and podocyte apoptosis in diabetic kidney.

It was reported that MafB was essential for differentiation and foot process formation of podocytes [14]. In our study, miR-320 suppressed the expression of MafB, which led to podocyte loss and kidney dysfunction in db/db mice. Moreover, the kidbey function was even worsened in the miR-320a overexpressed db/db mice with rAAV-miR-320a intravenous injection, featured with aggravated mesangial expansion and increased proteinuria, serum creatinine, and BUN levels. In contrary, both knockdown of miR-320a and re-expression of MafB alleviated podocyte injury as well as renal dysfunction in DN. Therefore, our data suggest that the miR-320a/MafB plays an adverse role via promoting hyperglycemia-induced podocyte loss and kidney dysfunction in DN.

A recent study indicated that Gpx3, an antioxidative stress enzyme, was produced by glomerular epithelial cell and interacted with podocin in kidney [60]. Moreover, Maf (c-Maf), a homolog of MafB, was a transcriptional regulator of Gpx3, which modulated the antioxidative pathway in the renal proximal tubule [61]. In the current study, we found that both the mRNA and protein expressions of Gpx3 was significantly reduced in diabetic glomeruli, which was further decreased by miR-320a overexpressed in db/db mice. Our data also showed that restoration of Mafb expression in db/db mice and downregulation of miR-320a in cultured HG-treated podocytes could increase the level of Gpx3 and ameliorate podocyte loss. Collectively, miR-320a/MafB suppressed the expression of Gpx3 in DN.

Expressed on the membrane of podocytes, Nephrin is the key composition of the glomerular slit diaphragm and critical in preventing proteinuria [6, 62]. Hyperglycemia resulted in the loss of Nephrin and may cause proteinuria in human DN and STZ-induced diabetic kidney dysfunction rats [60]. Moreover, it was reported that MafB stimulated Nephrin transcription through binding to the MARE within the proximal promoter of the Nephrin gene [19]. Our data showed that the loss of MafB suppressed Nephrin expression in podocytes of DN in vivo. In addition, upregulation of MafB by miR-320a inhibitor increased the level of Nephrin in HG-treated podocyte cells. Thus, miR-320a/MafB inhibited the expression of Nephrin in DN.

In summary, here we showed that miR-320a played a detrimental role in diabetic nephropathy. This effect was induced by suppressing slit-diaphragm proteins and antioxidative enzymes via inhibiting MafB in podocytes. Moreover, miR-320a could be a therapeutic target in diabetic nephropathy.

## METHODS

### Reagents

Fetal bovine serum (FBS), DMEM and RPMI 1640 were purchased from GIBCO (Grand Island, NY). Lipofectamine 2000 (Lipo 2000) reagent was from Invitrogen (Carlsbad, CA). The primers of human miR-320a and U6, human miR-320a mimics, human miR-320a inhibitor and their controls were provided by RiboBio (Guangzhou, China). Real-time PCR Primers of mRNA were synthesized by BGI Tech (Shenzhen, China). Antibodies against GFP (Cat No: AE012) was from Abclonal (Wuhan, China). Anti-Gpx3 (Cat No: AF-4199) and Nephrin (Cat No: AF3159) were from R&D System (Minneapolis, MN). Anti-GAPDH (Cat No: sc-32233) and anti-Desmin (Cat No: sc-65983) were from Santa Cruz Biotech (Dallas, TX). Anti-WT1 (Cat No: sc-7385) was from Santa Cruz Biotech (Dallas, TX). Anti-Ago2 (Cat No: H00027161-M01) was from Novus Biologicals (Littleton, CO). Anti-podocin (Cat No: 20384-1-AP) was from proteintech (Wuhan, China). Prestained protein markers, horseradish peroxidase (HRP)-conjugated secondary antibodies, Alexa Fluor 594 donkey anti-rabbit IgG (H+L) antibody (Cat No: A-21207), Alexa Fluor 594 donkey anti-mouse IgG (H+L) antibody (Cat No: A-21203) and enhanced chemiluminescence reagents were from Thermo Fisher Scientific (Rockford, IL). Polyvinylidene difluoride (PVDF) membranes were from Millipore (Darmstadt, Germany). FITC-phalloidin (Cat No: P5282) and other chemical reagents were purchased from Sigma-Aldrich Company (Shanghai, China).

### Preparation of recombinant adenoassociated virus (rAAV)

To manipulate the expression of miR-320a and MafB in vivo, we employed the rAAV system (type 9) which was a kind gift from Dr. Xiao Xiao (University of North Carolina at Chapel Hill). For the expression of miR-320a and miR-320a TuDs, oligonucleotides were designed as miR-random, miR-320a, miR-320a TuDs according to the mature sequence of hsa-miR-320a provided by miRBase (Accession: MIMAT0000510, Supplementary Table 2). The sequence of miR-random was provided by RiboBio (Guangzhou, China). The rAAVs were packaged in human embryonic kidney 293 (HEK293) cells and purified as described previously [63].

### Animals

The Institutional Animal Research Committee of Tongji Medical College approved all protocols. The investigation corresponded with the US National Institutes of Health guidelines for the Care and Use of Laboratory Animals. Male db/db mice and control mice, both of which were on C57BL/Ks, were supplied by Model Animal Research Center of Nanjing University (Nanjing, China).

C57BL/Ks mice were randomly divided into following groups (control, rAAV-miR-Random, rAAV-miR-320a, rAAV-miR-320a TuDs and rAAV-miR-320a + rAAV-MafB, n ≥ 8 for each group), and they were treated with corresponding rAAVs (1×10^11^ virions particles) via vein injection at the age of 8 weeks. Mice were sacrificed at the age of 10 weeks or 16 weeks, and tissue samples were collected, snap frozen in liquid nitrogen and stored at 80°C or fixed with formalin for further experiments.

Db/db mice were randomly divided into five groups (control, rAAV-miR-Random, rAAV-miR-320a, rAAV-miR-320a TuDs and rAAV-miR-320a + rAAV-MafB, n ≥ 8 for each group), and they were treated with corresponding rAAVs (1×10^11^ virions particles) via vein injection at the age of 8 weeks. Mice were sacrificed at the age of 24 weeks, and tissue samples were collected, snap frozen in liquid nitrogen and stored at 80°C or fixed with formalin for further experiments.

### Blood and urine biochemistry

Fasting blood glucose was measured by Glucose LiquiColor Test (Stanbio Laboratory, Boerne, TX) every 2 weeks. 24-hour urine volume of each animal was collected using metabolic cage system every 4 weeks. Serum creatinine was detected using the Creatinine Assay Kit (BioAssay System, CA) with an improved Jaffe method, while BUN was detected using the Urea Assay Kit (BioAssay System, CA) with an improved Jung method. Urine albumin was detected using the mouse albumin ELISA kit (Bethyl Laboratories, Montgomery, TX).

### Histology and immunohistochemical staining

Paraffin-embedded mouse kidney tissues were cut into 4-mm-thick sections and stained with PAS (Abcam, Shanghai, China) for histopathological examination under light microscopy. Paraffin-embedded sections were stained with anti-Desmin as described previously [64]. For immunofluorescence analysis, paraffin-embedded sections were stained with anti-Nephrin, while MPC5 cells were stained with FITC-phalloidin. Sections and cells were observed under the laser scanning confocal microscope (Olympus, FV500-IX71, Tokyo, Japan).

### Podocyte number counting

Paraffin-embedded kidney sections were immune-stained with a monoclonal antibody against WT1, and then detected by the avidin-biotin-peroxidase complex staining technique using a Histofine Kit (Nichirei, Tokyo, Japan). The number of WT1-positive cells was counted in 20 glomeruli of each section at × 400 magnification, and the mean number was recorded as the podocyte number in each sample.

### Quantification of ROS production

Dihydroethidium (DHE; Invitrogen, Carlsbad, CA) was applied to kidney frozen sections (6μm) at 40 μmol for 30 minutes. Sections was detected under a Nikon DXM1200 fluorescence microscope, and fluorescence intensity were analyzed with the Image-Pro software (Media Cybernetics, Rockville, MD)

### Cell culture, transfection, and treatments

Conditionally immortalized podocytes (MPC5), established by Peter Munde [65], were purchased from Peking Union Medical College Basic Medical Sciences Cell Resource Center (Beijing, China). Undifferentiated MPC5 were cultured at 33°C in RPMI 1640 containing 10% fetal bovine serum and 50 IU/ml of recombinant murine IFN-γ. The cells were transferred to 37°C in RPMI 1640 containing 5% FBS without IFN-γ for 10–14 days to induce differentiation. These MPC5 cells were stained with podocyte markers, Nephrin, WT1 and podocin, by immunofluorescence assays to identify the characteristics of murine podocytes (Supplementary Figure 7).

HEK293 cells were from American Type Tissue Collection and were cultured in DMEM with 10% FBS. Cells were grown in a 95% air, 5% CO_2_ atmosphere. Cells were transfected with human miR-320a mimics (100 nM, similarly hereinafter), human miR-320a inhibitor (100 nM) or their negative control (100 nM) respectively using Lipo 2000 reagent according the manufacturer’s protocol. After transfection, MPC5 cells were treated with normal glucose (NG, 5 mmol/L D-glucose) or high glucose (HG, 30 mmol/L D-glucose) for 48 h and then collected.

### RNA isolation and detection

We performed total RNA extraction from frozen tissues or cells and plasma using TRIzol Reagent (Invitrogen, Carlsbad, CA) and TRIzol LS Reagent (Invitrogen, Carlsbad, CA), respectively, and quantified miRNA or mRNA expression levels by real-time PCR according to the manufacturer’s instructions with an ABI 7900HT Detection system (Applied Biosystems, Foster City, CA). Each sample has three replicates. GAPDH was used as endogenous control to mRNA, while U6 small nuclear RNA was used as endogenous control to miRNA. The primer sequences were listed in Supplementary Table 2.

### Western blot

Protein samples from cell and mice kidney lysates (30 μg) were quantified using the bicinochoninic acid assay kit (BOSTER, Wuhan, China). Then samples were resolved by SDS-PAGE, transferred to PVDF membranes, and incubated with primary and secondary antibodies. Amount of proteins was determined from the blot using ImageJ (National Institutes of Health Software, Bethesda, MD) and normalized to the GAPDH level.

### Target prediction of miRNA

MiR-320a target prediction was performed with bioinformatic prediction web sites miRWalk (http://zmf.umm.uni-heidelberg.de/apps/zmf/mirwalk2/miRretsys-self.html) and RNAhybrid (https://bibiserv.cebitec.uni-bielefeld.de/rnahybrid/).

### Co-immunoprecipitation with anti-Ago2 antibody

Podocytes were lysed 24 hours after transfection with miR-320a mimics or control, and then immunoprecipitated with anti-Ago2 antibody or anti-IgG as described previously [37, 66, 67]. Ago2 immunocomplexes were extracted with TRIzol, and the levels of MafB mRNA were quantified by real-time PCR. Lysates of renal cortex from differently treated db/db mice were also analyzed.

### Dual luciferase assay

400 ng of pMIR-MafB 3′-UTR, pMIR- MafB 3′-UTR mutant, or the empty vector was transfected into HEK293 cells accompanied with 40 ng of pRL-TK plasmid (Promega, Madison, WI), respectively. Meanwhile, miR-320a mimics or control was co-transfected with those reporter plasmids at a final concentration of 100 nM. Forty-eight hours later, luciferase activity was detected using Dual-Luciferase Reporter Assay System (Promega, Beijing, China) and normalized by measuring renilla luciferase activity.

### TUNEL assay

Apoptotic cells were detected using in situ cell death detection kits (Roche Diagnostics GmbH, Mannheim, Germany) according to the manufacturer’s instructions. For identification of apoptotic podocytes, the kidney paraffin-embedded sections were co-stained with WT1 antibody. For quantitative analysis, the degree of apoptosis was estimated by the mean number of TUNEL positive cells per 100 glomerular sections.

### Statistics

Data was analyzed using a one-way ANOVA and a t-test with Dunnett comparison using Prism (version 6; GraphPad Software, La Jolla, CA) and expressed as the Mean ± SEM. Values were considered significantly different if *P <* 0.05.

## Supplementary Material

Supplementary Figures

Supplementary Tables
