# Correction for: MiR-320a induces diabetic nephropathy via inhibiting MafB

**DOI:** 10.18632/aging.204441

**Published:** 2022-12-15

**Authors:** Mengying He, Jin Wang, Zhongwei Yin, Yanru Zhao, Huiying Hou, Jiahui Fan, Huaping Li, Zheng Wen, Jiarong Tang, Yan Wang, Dao Wen Wang, Chen Chen

**Affiliations:** 1 Division of Cardiology and Hubei Key Laboratory of Genetics and Molecular Mechanisms of Cardiological Disorders, Tongji Hospital, Tongji Medical College, Huazhong University of Science and Technology, Wuhan, 430030, China

**Keywords:** miRNA, podocyte, diabetic nephropathy

**This article has been corrected:** The authors found that some of the representative images in **Figures 6O** and **6P** were duplications of the images in **Figures 2M** and **2N**, respectively. They replaced the incorrect “Merged” image of ROS detected with the DHE probe in frozen kidney sections from the C57BL/Ks group in **Figure 6O** with the correct image from the original experiments. In **Figure 6P**, all three stainings (TUNEL, WT1, Hoechst) and the Merged images of apoptotic glomerular cells in diabetic glomeruli from db/db mice treated with miR-320a+MafB were replaced. The authors stated that since the corresponding quantitative analyses are correct, this correction does not affect the conclusions of the published research.

New **Figure 6 panels O** and **P** are presented below.

**Figure 6:**
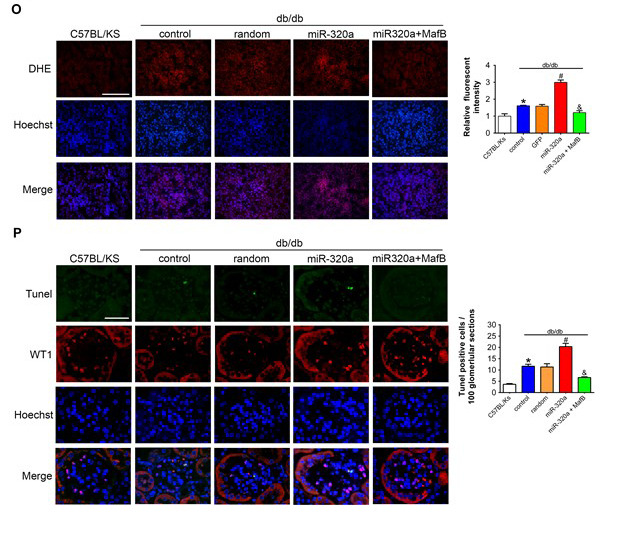
**MafB restoration attenuated miR-320a induced kidney injury in diabetes.**... (**O**) Representative images of ROS detected by DHE probe in frozen kidney sections. Scale bar, 200 μm. (**P**) Typical images of apoptotic glomerular cells in diabetic glomeruli. Green, TUNEL; Red, WT1; Blue, Hoechst. Scale bar, 50 μm. Data are expressed as mean ± SEM, n=8, *P<0.05 versus C57BL/Ks, #P<0.05 versus db/db control, &P<0.05 versus db/db control.

