# Supplementary material for: MiR-320a induces diabetic nephropathy via inhibiting MafB
Source: Aging (Albany NY). 2019 May 17;11(10):3055–79. doi: 10.18632/aging.101962 (PMC6555468; doi:10.18632/aging.101962)
Supplement: Supplementary Figures [file aging-11-101962-s001.pdf]

SUPPLEMENTARY TABLES

Supplementary Table 1. Selectively up-regulated genes in glomeruli of diabetic mice determined by fold changes.

Gene symbol	P Value	Fold changes	MFE* (kcal/mol)	Conservation#
Tap1	0.004701	0.104298	0	
Fgfbp1	0.001049	0.192311	0	
Inmt	0.000365	0.241215	-23.5	
Psmb8	0.004235	0.241593	-25	No
Bdh2	0.009131	0.280667	-23.8	
Pcp4l1	0.000517	0.285062	0	
Cyp2s1	0.004148	0.29442	-21	
Tlr7	0.000789	0.305639	-18.6	
Il33	0.001501	0.350226	-16.3	
Fndc5	0.005792	0.352135	0	
Slc2a6	0.000943	0.355426	-28.9	Yes
Ntf3	0.005003	0.364439	0	
Fxyd6	6.27E-05	0.366654	0	
Fam213b	0.002963	0.371535	-26	No
Ctsh	0.006695	0.375822	-22.2	
Thrsp	0.00554	0.378472	0	
Cxcl12	0.000455	0.396373	-17.3	
Mafb	0.002585	0.397118	-26.7	Yes
Bmpr1b	0.009987	0.398378	-17.7	
Dzank1	0.000798	0.413896	-26.4	No
Idnk	0.002582	0.420367	-23.5	
Met	0.005306	0.42376	-23	
Stxbp6	0.002381	0.425538	-17.9	
Pik3ip1	0.005499	0.427406	-13.2	
Lbp	0.002778	0.432382	0	
Pgf	0.000209	0.432481	-15.6	
Lztfl1	0.006218	0.434302	-26.5	No
Rtp4	0.00853	0.442181	0	
Dkk3	0.000816	0.442939	-21.2	
Tnfsf10	0.00253	0.442997	-19.5	
Folr1	0.007362	0.448012	0	
Itgb3bp	0.001552	0.453235	0	
Ptprr	0.000699	0.453238	-23.9	
Dgat2	0.003021	0.454399	-17.7	
Ssbp2	0.003343	0.45716	-20	
Uba7	0.007952	0.458067	-26.5	Yes
Rrad	0.000098	0.461464	0	
Spon1	0.001218	0.464165	-19.4	
Enpp1	0.009829	0.464825	-24.3	
Cmbl	0.009802	0.472668	-25.9	No
Fmod	0.005258	0.475254	-20.5	
Snrpd3	0.003166	0.479955	-32.2	No
Kcnb1	0.000854	0.480044	-26.4	No

Sulf1	0.004135	0.481424	-27.3	No
Usp15	0.002286	0.483005	-23	
Pdk2	0.005547	0.483049	-23.1	
Tesk2	0.004593	0.483682	-16.5	
Vdr	0.006662	0.485163	-19	
Mrps27	0.000217	0.485782	-15.8	
Cyp26b1	0.004756	0.487043	-19.9	
Adat2	0.003494	0.49244	-32.1	No
Dhps	0.000819	0.496206	-24.6	No

* MFE, minimum free energy of has-miR-320a and its target genes were evaluated using RNAhybrid. And the genes were selected when the mfe were less than or equal to -25.0 kcal/mol.
# The conversation of sequence coding for the selected genes were analyzed among human, mouse and rat.

Supplementary Table 2. The oligonucleotides sequences designed for the expression of miR-random, miR-320a and miR-320a TuDs.

	Oligonucleotides sequence
miR-random	5′- GATCCTTTGTACTACACAAAAGTACTGTTCAAGAGACAG TACTTTTGTGTAGTACAAACCGC-3′
miR-320a	5′- AGCTTTCGCCCTCTCAACCCAGCTTTT TTCAAGAGAAAA AGCTGGGTTGAGAGGGCGACCGC-3′
miR-320aTuDs	5′- GACGGCGCTAGGATCATCAACTCGCCCTCTCAAATCTCC CAGCTTTTCAAGTATTCTGGTCACAGAATACAACTCGCCCTCTCAAATCTCCCAGCTTTT CAAGATGATCCTAGCGCC GTCTTTTTT-3′

Supplementary Table 3. The primer sequences designed for real-time PCR.

	Forward primers	Reverse primers
MafB	5′-TGAGCATGGGGCAAGAGCTG-3′	5′-CCATCCAGTACAGGTCCTCG-3′
Nephrin	5′-GCCACCACCTTCACACTGAC-3′	5′-AGACCACCAACCGCAAAGA-3′
Gpx3	5′-GATGTGAACGGGGAGAAAGA-3′	5′-CCCACCAGGAACTTCTCAAA-3′
GAPDH	5′- GAGTGTTTCCTCGTCCCGTAG-3′	5′- GAAGGGGTCGTTGATGGCAA-3′
