# Supplementary material for: MiR-320a induces diabetic nephropathy via inhibiting MafB
Source: Aging (Albany NY). 2019 May 17;11(10):3055–79. doi: 10.18632/aging.101962 (PMC6555468; doi:10.18632/aging.101962)
Supplement: Supplementary Tables [file aging-11-101962-s002.pdf]

SUPPLEMENTARY FIGURES

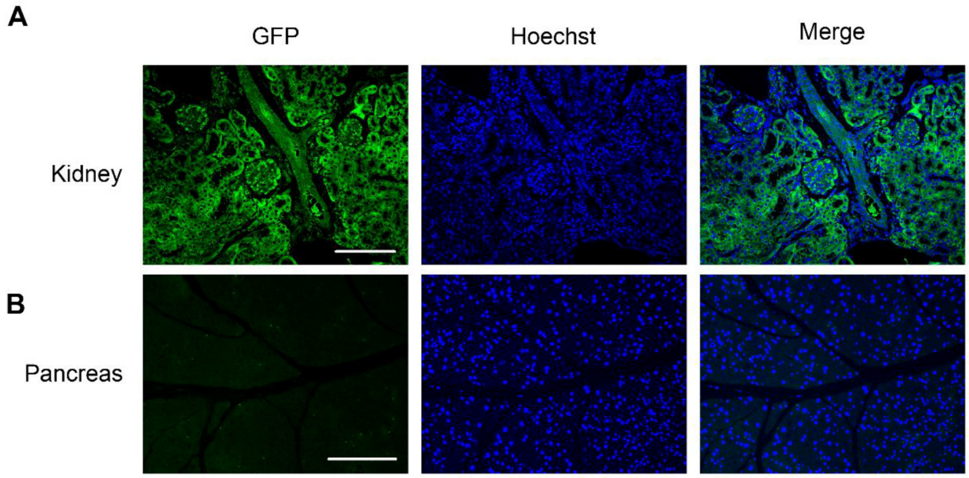

Supplementary Figure 1. rAAV-GFP succesfully transfected in the murine kidney. Representative images of immunofluorescence staining for GFP in (**A**) kidney and (**B**) pancreas. Scale bar, 200 µm.

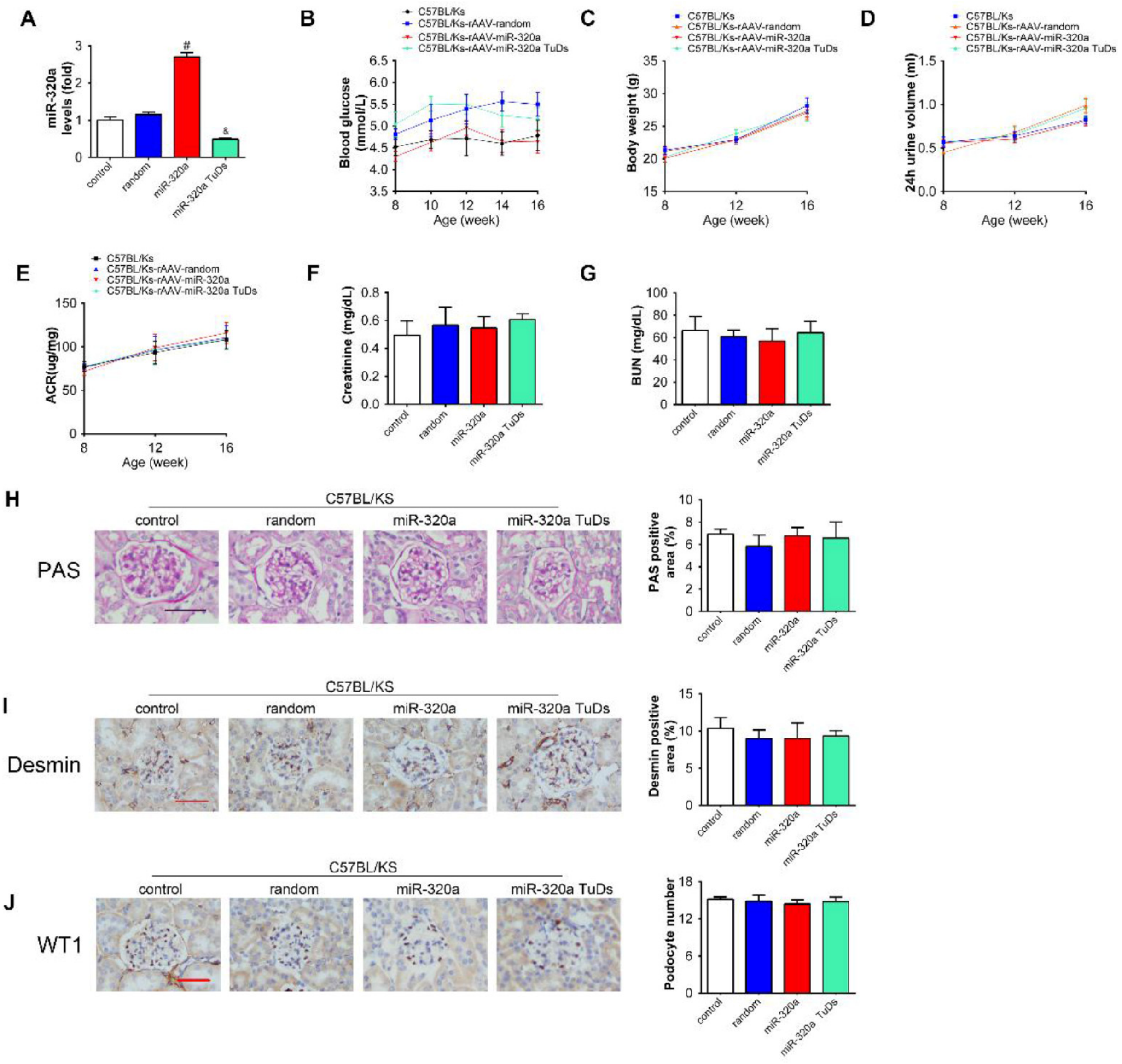

Supplementary Figure 2. Overexpression of miR-320a didn't change renal function in normal C57BL/Ks mice. (**A**) Relative miR-320a expression in renal cortex measured by real-time PCR. (**B**) Blood glucose was detected every 2 weeks. (**C**) Body weight, (**D**) 24h urine volume and (**E**) urinary ACR was determined every four weeks since the age of 8 weeks. (**F**) Serum creatinine and (**G**) BUN were detected at the age of 16 weeks. (**H**) Representative images of PAS staining of kidneys from differently treated C57BL/Ks mice. Scale bar, 50 μm. (**I**) Representative images of immunohistochemical staining of Desmin. Scale bar, 50 μm. (**J**) Typical images of WT1-stained glomeruli and average number of WT1-stained nuclei calculated per glomerular sections. Scale bar, 50 μm. Data are expressed as mean ± SEM, n=8, #P<0.05 versus control, &P<0.05 versus control.

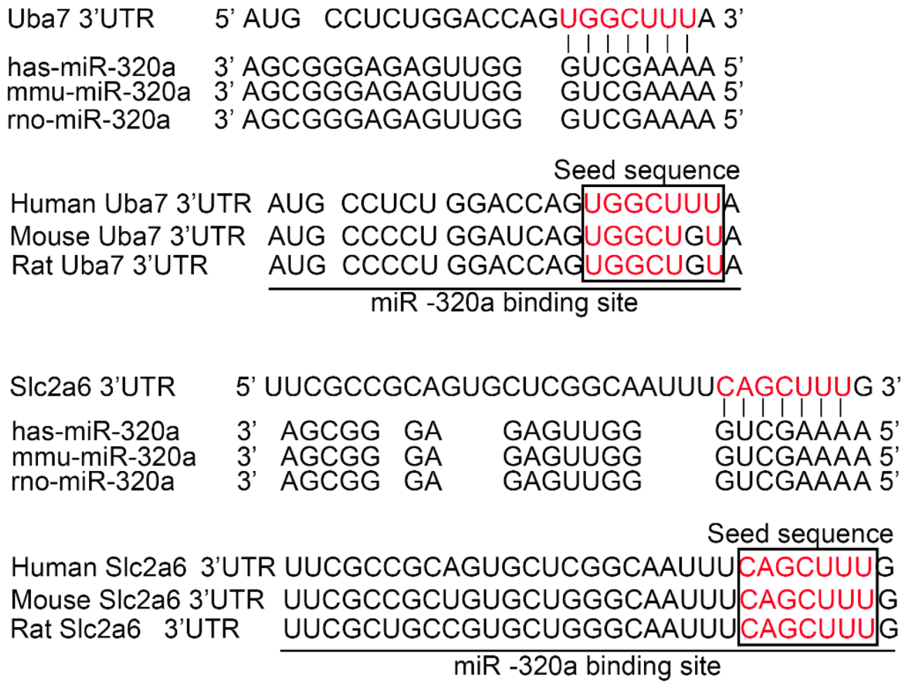

Supplementary Figure 3. miR-320a and the 3′-UTR of predicted genes among three species.

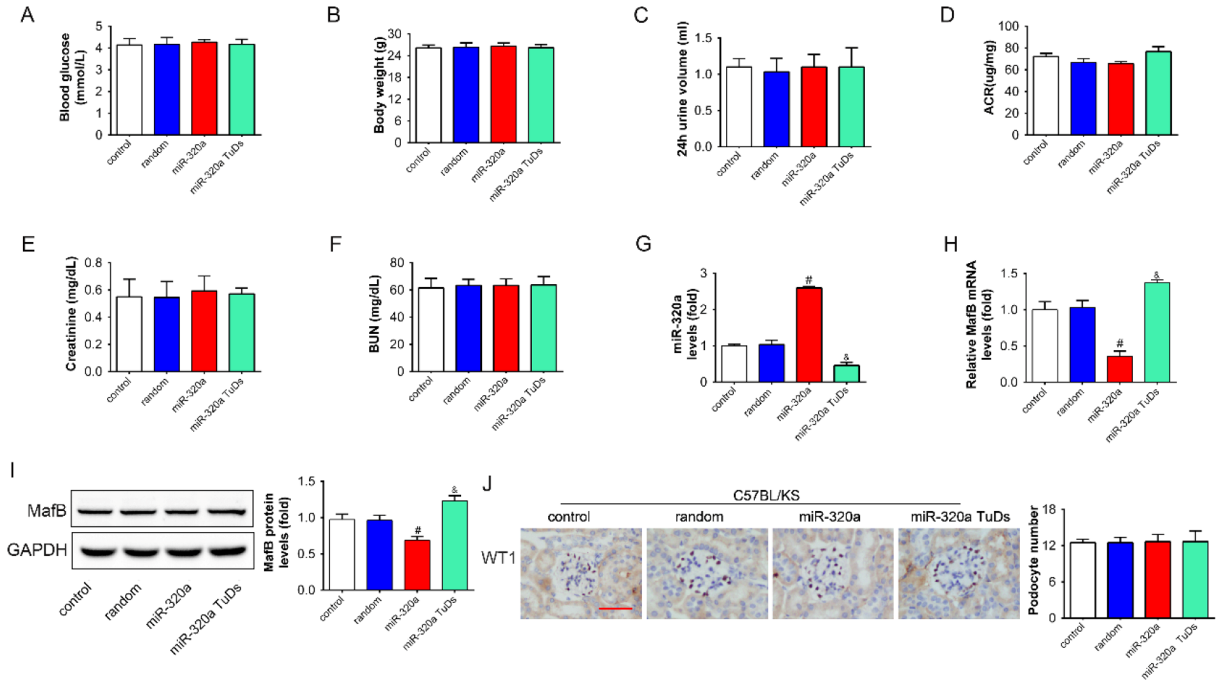

Supplementary Figure 4. (A) Blood glucose, (B) Body weight, (C) 24h urine volume and (D) urinary ACR, (E) Serum creatinine and (F) BUN were detected at the age of 10 weeks. Relative (**G**) miR-320a, MafB (**H**) mRNA and (**I**) protein levels in differently treated C57BL/Ks mice. (**J**) Typical images of WT1-stained glomeruli and average number of WT1-stained nuclei calculated per glomerular sections. Scale bar, 50 μm. Data are expressed as mean ± SEM, n=8, #P<0.05 versus control, &P<0.05 versus control.

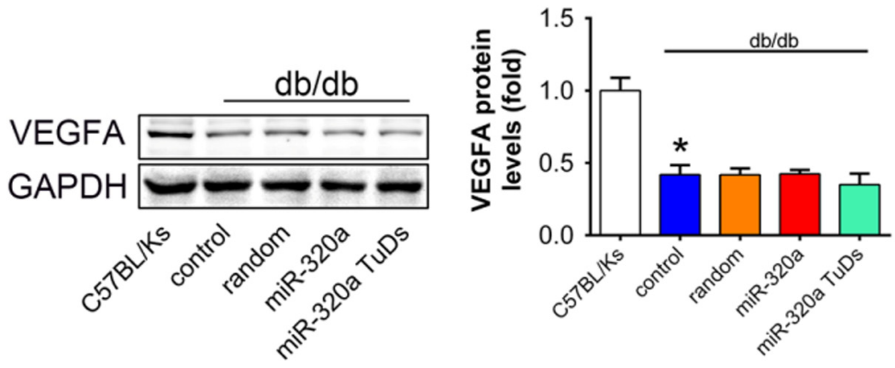

Supplementary Figure 5. The VEGFA protein level in differently treated db/db mice. VEGFA protein levels in the kidney of differently treated db/db mice detected by western blot. Data are expressed as mean ± SEM, n=8, *P<0.05 versus C57BL/Ks, #P<0.05 versus db/db control, &P<0.05 versus db/db control.

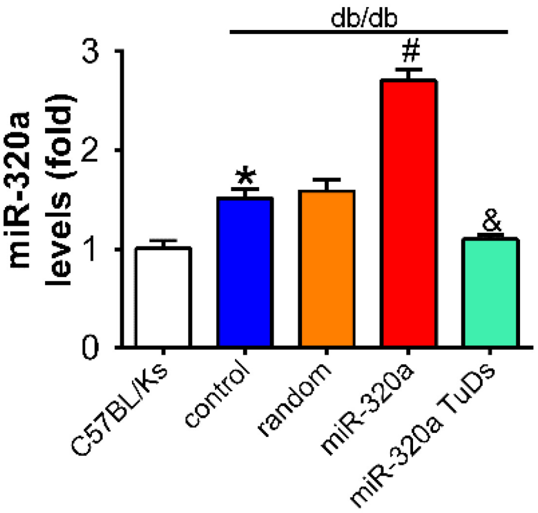

Supplementary Figure 6. The circulating miR-320a levels in db/db mice. Relative circulating miR-320a levels in db/db mice measured by real-time PCR. Data are expressed as mean ± SEM, n=8, *P<0.05 versus C57BL/Ks, #P<0.05 versus db/db control, &P<0.05 versus db/db control.

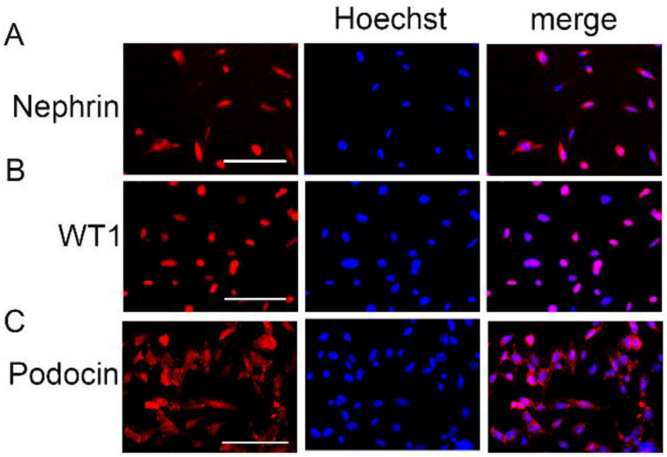

Supplementary Figure 7. MPC5 cells retain the characteristics of murine podocytes. Representative images of immunofluorescence staining for (**A**) Nephrin, (**B**) WT1 and (**C**) podocin..Scale bar, 25 μm.
